# Draft Genomes of Symbiotic* Frankia* Strains AgB32 and AgKG'84/4 from Root Nodules of *Alnus Glutinosa* growing under Contrasted Environmental Conditions

**DOI:** 10.7150/jgen.75779

**Published:** 2022-08-08

**Authors:** Philippe Normand, Petar Pujic, Danis Abrouk, Spandana Vemulapally, Trina Guerra, Camila Carlos-Shanley, Dittmar Hahn

**Affiliations:** 1 Université Claude-Bernard Lyon 1, Université de Lyon, UMR 5557 CNRS Ecologie Microbienne, Villeurbanne, Cedex 69622, France.; 2 Texas State University, Department of Biology, 601 University Drive, San Marcos, TX 78666, USA.

**Keywords:** *Frankia*, Actinorhizal symbiosis, genome, nitrogen-fixing frankiae, biosynthetic gene clusters

## Abstract

The genomes of two nitrogen-fixing *Frankia* strains, AgB32 and AgKG'84/4, were isolated from spore-containing (spore+) and spore-free (spore-) root nodules of *Alnus glutinosa*, but they did not sporulate upon reinfection. The two strains are described as representatives of two novel candidate species*.* Phylogenomic and ANI analyses indicate that each strain represents a novel species within cluster 1, with genome sizes of 6.3 and 6.7 Mb smaller than or similar to those of other cultivated *Alnus*-infective cluster 1 strains. Genes essential for nitrogen-fixation, clusters of orthologous genes, secondary metabolite clusters and transcriptional regulators analyzed by comparative genomic analyses were typical of those from *Alnus*-infective cluster 1 cultivated strains in both genomes. Compared to other cultivated *Alnus*-infective strains with large genomes, those of AgB32 and AgKG'84/4 had lost 380 or 409 genes, among which one *hup* cluster, one *shc* gene and the *gvp* cluster, which indicates genome erosion is taking place in these two strains.

## Introduction

Bacteria classified in the genus *Frankia* constitute a heterologous group of filamentous soil bacteria that can trigger the development of symbiotic root nodules on a range of host plants belonging to 25 genera of perennial, dicotyledonous, angiosperms 1-3. Isolates have been classified into four distinct clusters, among which three comprise strains that fix atmospheric nitrogen (N_2_), either in pure culture or in nodules, while cluster 4 frankiae for the most part do not fix N_2_, except for one strain, and are often unable to fulfil Koch's postulates 4, 5. Cluster 1 comprises strains infective on *Alnus* and *Casuarina*, with currently four described species and two candidate species 6. The species have type strains deposited in culture collections as *Frankia alni* ACN14a^T^ 7, *F. torreyi* CpI1^T^ 8, *F. casuarinae* CcI3^T^ 7 and *F. canadensis* ARgP5^T^ 9. Candidate species represent uncultured *Frankia* populations in root nodules of host plants, i.e. Candidatus *F. nodulisporulans* AgTrS, AgUmASt1 and AgUmASH1 10 and Candidatus *F. alpina* AiOr, and AvVan 10 that have resisted all attempts at culture.

Several published works on genus *Frankia* using sub-cluster, OTU, group and genomospecies assignments did provide grounds permitting to affirm that cluster 1 is probably much more diverse than the four species and two candidate species described so far 4, 11-14. This statement is supported by recent genome analyses of strains Ag45/Mut14 and AgPM24 as representatives of a yet undescribed species 15, and by comparative sequence analyses of amplicons of an actinobacteria-specific insertion in the 23S rRNA genes of frankiae that identified several strains clustering together but that are distinct from type strains of cluster 1 16. Strains AgB32 and AgKG'84/4 are two such strains, isolated from root nodules of *Alnus glutinosa* growing under contrasted environmental conditions at two locations in Germany about 350 km apart. Strain AgB32 was isolated from spore[+] root nodules of *Alnus glutinosa* of a forest ecotype that was interspersed with oak (*Quercus robur*) in an established riverside forest on a wet, but well aerated sandy loam in Bad Bentheim, Germany (52.320319, 7.159997) 17. Strain AgKG'84/4 was isolated from spore[-] root nodules of *A. glutinosa* of the pioneer ecotype growing in a pure stand at a lake shore marsh in water-logged soil rich in organic material in Krems II-Goels, Germany (53.989103, 10.360772) 17. Both strains had previously been identified as members of cluster 1, representing a subcluster designated as subgroup I 18 or cluster 1d 16. In order to assess the viability of the previous amplicon-based analysis and to potentially amend and refine the species diversity of cluster 1 frankiae, we used whole genome sequence analyses trying to affirm the potential of strains AgB32 and AgKG'84/4 for the description of new species.

## Materials and Methods

### Sample preparation

*Frankia* strains AgB32 and AgKG'84/4 were grown from stocks preserved in 20% vol/vol glycerol at -80 °C since 2003 in Defined Propionate Medium (DPM) containing propionate and NH_4_Cl as C and N source, respectively (19), at 30 °C for two weeks. Cells were harvested by centrifugation (15,000 × g, 5 min) and homogenized through sonication (10 s at 20% output in a S-450 sonifier, Branson Ultrasonics, Danbury, CT) 20. DNA was extracted from cell pellets after an additional centrifugation step using the SurePrep^TM^ Soil DNA Isolation Kit (Fisher Scientific, Houston, TX) 21, and concentrations measured with a Qubit^®^ 2.0 Fluorometer (Life Technologies, Carlsbad, USA). Library preparation and sequencing using the Illumina tagmentation protocol and the NextSeq Illumina platform (2 × 150 bp) using standard protocols were done at the Microbial Genomics Sequencing Center, Pittsburgh, PA, USA.

### Genome assembly

Default settings of fastp were used to filter and trim sequence reads 22, with reads with average %GC<54 removed using bbduk (https://jgi.doe.gov/data-and-tools/bbtools/bb-tools-user-guide/). SPAdes 3.13.0 was used to assemble genomes 23 and QUAST to check their quality 24. Genome completeness was estimated using the lineage workflow (lineage_set) CheckM v1.0.18 with default values 25.

### Comparative genomic analysis

Assembled genomes of strains AgB32 and AgKG'84/4 as well as *Frankia* genomes of type strains of all described species and other selected genomes were selected for Average Nucleotide Identity (ANI) comparisons 26 using the pyani platform with the b (Blast) setting (27; https://pyani.readthedocs.io). Genomes were further analyzed on the Mage platform 28 to compute clusters of orthologous genes (COGs) 29, to identify secondary metabolite clusters through antiSMASH 30 and to identify genes specific to or lost in the new genomes. A MASH distance matrix 31 was used to construct a phylogenetic tree using a rapid neighbour joining algorithm 32 on the Mage platform.

## Results

### Characteristics of the two *Frankia* genomes

The genomes of the two strains AgB32 and AgKG'84/4 were considered complete given their CheckM scores of 99.59% and 98.05%, respectively. The N50 were 55 309 and 112 139, respectively and the total length were 6 667 069 and 6 426 475. They were considered pure with contamination indices of 1.09 and 2.37, respectively. Genomes of AgB32 and AgKG'84/4 harbored 214 and 1,305 contigs with the largest contig being 223 506 nt and 54 816 nt, respectively. Their GC contents of 72.23 and 71.88% for AgB32 and AgKG'84/4, respectively (Table 1).

### Phylogenetic analysis of *Frankia* spp

**Figure 1:**
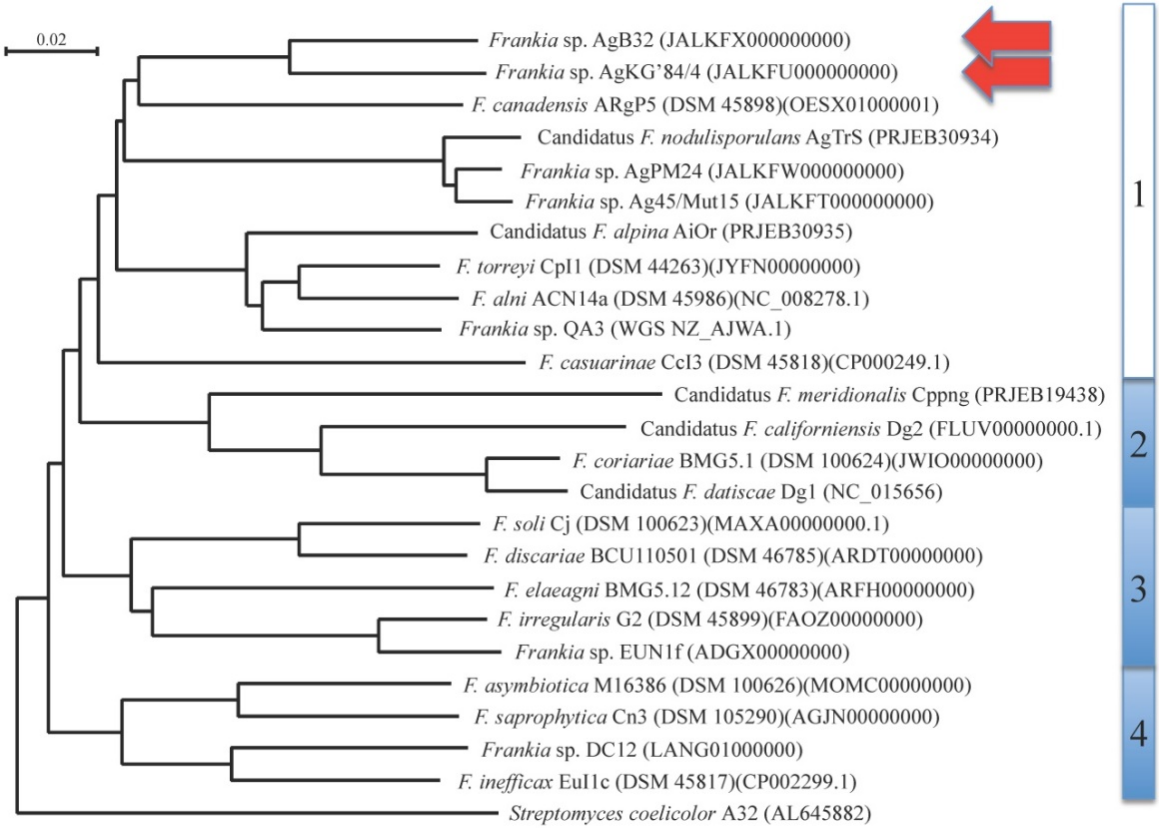
Phylogenetic tree based on comparative sequence analyses of complete genomes of *Frankia* species and candidate species, using *Streptomyces coelicolor* (AL645882) as outgroup. *Frankia* clusters 1 to 4 are indicated on the right. Scale units are substitutions per site. The two genomes described in the present study have red arrows.

**Figure 2:**
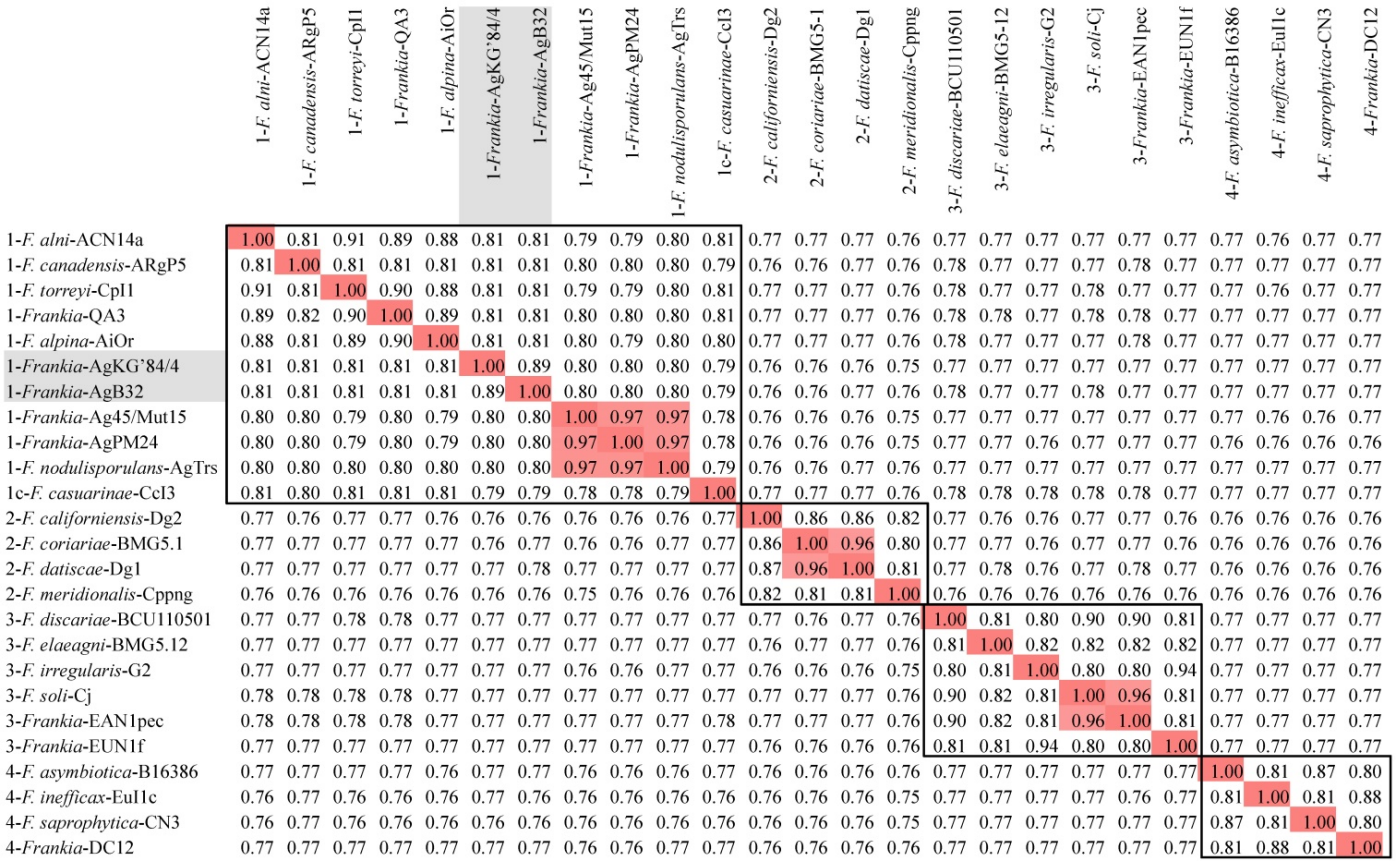
Heatmap matrix of Average Nucleotide Identity (ANI) comparisons (in percent) for the *Frankia* genomes of type strains of described species using the pyani platform with the b (Blast) setting 27; https://pyani.readthedocs.io). The two genomes described in the present study are highlighted in grey. Those ANI values above the 95% threshold are highlighted in red. ANI values of clusters are boxed.

A phylogenetic tree generated from the MASH matrix with *Frankia* genomes of type strains revealed that the closest strains to AgB32 and AgKG'84/4 were members of cluster 1 (Figure 1). Average nucleotide identity (ANI) between strains AgB32 and AgKG'84/4 was 89%, indicating that they belong to two separate genospecies (Figure 2). ANI values at or below 80% were obtained for both strains in comparison with *Frankia* genomes of type strains of all described species (Figure 2). The ANI values with other cluster 1 genomes ranged from 79% (CcI3) to 81% (ACN14a), while 76-77% values were obtained with cluster 2 genomes, and 77-78% with cluster 3 and 4 genomes (Figure 2).

### Analysis of functional genes in *Frankia* spp. isolates

All genes identified as playing a role in the symbiosis were found to be present in the genomes of AgB32 and AgKG'84/4, i.e. *nif, hup, suf, shc, cel, glx, bcsA* (Table 1). Furthermore, all genes that are more abundant in symbiotic lineages (clusters 1, 2 and 3) than in non-symbiotic lineages (cluster 4) (*sodF, geoA, argF, accA, rhbE, dctA, phdA*, *tgsA, ddnB*) were also recovered in AgB32 and AgKG'84/4 (Table 1). Conversely, *gvp* that codes for gas vesicle proteins, one of the two *shc* genes and one of the two *hup* clusters that are found in cluster 1 strains were not found in the two genomes while the symbiotic cluster was maintained 33.

The COG computation showed values for AgB32 and AgKG'84/4 characteristic of other* Alnus*-infective cluster 1 strains with a low number of categories “N” (Cell motility), and “P” (Inorganic ion transport and metabolism) (Table 2). These results are similar for the antiSMASH computation that showed AgB32 and AgKG'84/4 to have values characteristic of other* Alnus*-infective cluster 1 strains with a high number of T1PKS and NRPS (Table 3). T1PKS and NRPS typically code for antibiotics and a high number of such clusters is evocative of a good capacity for keeping other soil microbes at bay. The numbers of transcriptional regulators were on the whole comparable to other strains with a low number of ArsR, and LuxR regulators (Table 4).

A search for genes present in *F. alni* ACN14a, *Frankia* sp. QA3, *F. torreyi* CpI1 and *F. canadensis* ARgP5 but absent in AgB32 and AgKG'84/4 yielded 380 or 409 hits, respectively among which an alkane sulfonate, a acetyl/propionyl CoA carboxylase locus, an uptake hydrogenase locus, a dicarboxylate transporter, a Hup locus, the GVP locus, several transporters (Table S1). Conversely, there were 565 genes present in both AgB32 and AgKG'84/4 but absent in *F. alni* ACN14a, *Frankia* sp. QA3, *F. torreyi* CpI1 and *F. canadensis* ARgP5, of which about half (277) were of unknown function.

## Discussion

The genus *Frankia* has been scantily described for many years because of difficulties to isolate and grow frankiae in pure culture, a major prerequisite for the description of strains 34, 35. Some populations to this day have even resisted isolation attempts so far 36. Differentiation of isolates has also been hampered by the availability of few distinguishing features between populations 14. Starting in 2007, new developments in whole genome sequencing techniques have overcome these difficulties and resulted in the determination of genome sequences of three *Frankia* isolates 37, and ultimately even of uncultured *Frankia* populations in root nodules 38. Comparative analyses of whole genome sequences between *Frankia* populations have resulted in the description of twelve species and five candidate species for uncultured populations so far 6. These numbers were based on the availability of 37 genomes 39, a number that is increasing regularly 15, 40. Comparative sequences analyses of whole genomes and metrics such as ANI 26 or dDDH 41 are now used as foundation for the description of microbial genera, species and subspecies.

Members of the genus *Frankia* have been assigned into four clusters, numbered 1 to 4, within the genus 4. These assignments have proven quite solid over the years, with cluster 1 in particular found to remain coherent with all *Alnus*-infective symbiotic strains. Cluster 1c with *Casuarina*-infective strains remains at the root of this cluster with several distinguishing features such as the lack of vesicles in nodules, a host-derived hemoglobin protection against oxygen and a distinct host range 6. *Alnus*-infective symbiotic strains have been described initially on the basis of DNA/DNA homology as quite close to one another 14 but the full extent of diversity has slowly emerged with studies targeting new cultured strains and uncultured frankiae from specific environments 38, 42-47.

Genomes of *Alnus*-infective symbiotic strains have initially been found to be quite large at 7.5 Mb with several ancient duplicated genes such as the *shc* gene coding for the synthesis of hopanoid lipids 48, the *hup* genes coding for hydrogen uptake for the recycling of hydrogen derived from nitrogenase 33, the *cel* coding for cellulases 49, the *can* coding for the carbonic anhydrase necessary for feeding short chain fatty acids (SCFA) into the tricarboxylic acid (TCA) cycle or the *kor* genes coding for 2-oxoglutarate ferredoxin oxidoreductase that connects the TCA cycle with nitrogenase (with the nitrogen-fixation process) 50, 51. Some of these duplications have been found to be lost in lineages with smaller genome size as is the case for Ag45/Mut15 and AgPM24 15. It appears the genomes of strains AgB32 and AgKG'84/4 are also undergoing a parallel process of genome erosion. This process is similar with some of the genes lost in common such as *hup* but also other genes such as *shc* only lost in AgB32 and AgKG'84/4.

AgB32 and AgKG'84/4 are two distinct lineages with an ANI of 89%, well below the threshold of 95 set by Goris 26 to delineate species but markedly above the 80% average between other *Alnus*-infective cluster 1 species. This would indicate the two strains should constitute two distinct species yet sharing many features due to a recent common ancestry.

## Supplementary Material

Supplementary table.

## Figures and Tables

**Table 1:**
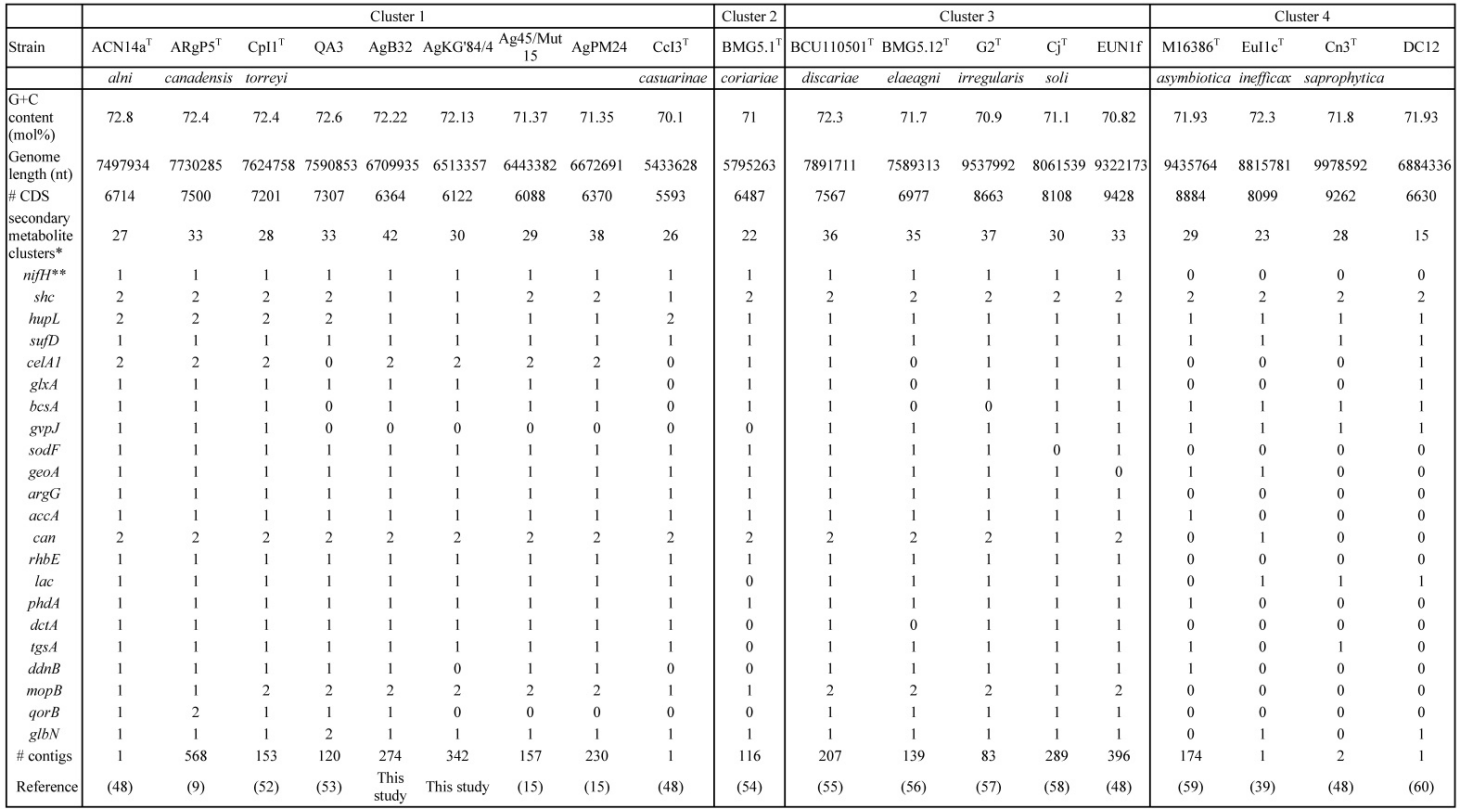
Basic genome characteristics (G+C%, genome length, number of CDS, number of secondary metabolite clusters, presence of selected genes, # of contigs and references) of *Frankia* strains AgB32 and AgKG'84/4 compared to those of type strains of *Frankia* species in clusters 1 to 4

**Table 2:**
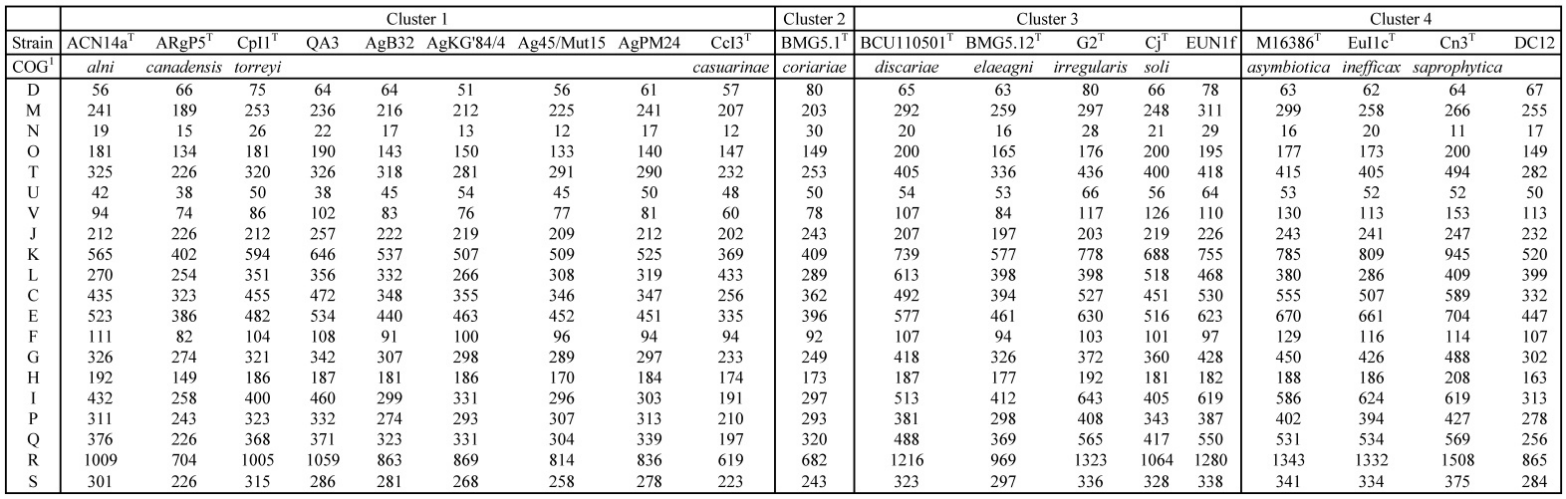
COG characteristics of *Frankia* strains AgB32 and AgKG'84/4 compared to those of type strains of *Frankia* species in clusters 1 to 4

^1^class: **D**: Cell cycle control, cell division, chromosome partitioning; **M**: Cell wall/membrane/envelope biogenesis; **N**: Cell motility; **O**: Posttranslational modification, protein turnover, chaperones; **T**: Signal transduction mechanisms; **U**: Intracellular trafficking, secretion, and vesicular transport; **V**: Defense mechanisms; **J**: Translation, ribosomal structure and biogenesis; **K**: Transcription; **L**: Replication, recombination and repair; **C**: Energy production and conversion; **E**: Amino acid transport and metabolism; **F**: Nucleotide transport and metabolism; **G**: Carbohydrate transport and metabolism; **H**: Coenzyme transport and metabolism; **I**: Lipid transport and metabolism; **P**: Inorganic ion transport and metabolism; **Q**: Secondary metabolites biosynthesis, transport and catabolism; **R**: General function prediction only; **S**: Function unknown.

**Table 3:**
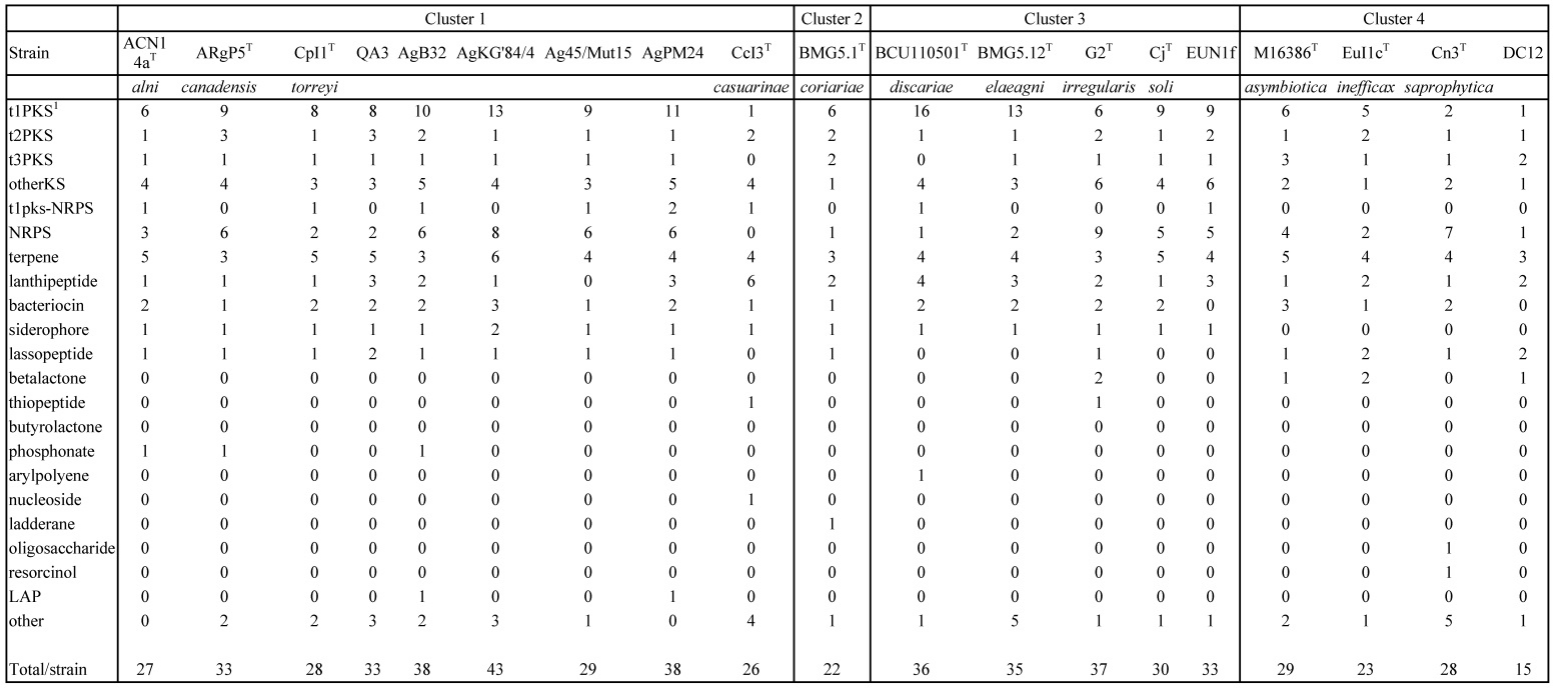
Number of secondary metabolites clusters (antiSMASH) of *Frankia* strains AgB32 and AgKG'84/4 compared to those of cultivated type strains of *Frankia* species in clusters 1 to 4

^1^tnPKS is type “n” Polyketide Synthase; NRPS is Non Ribosomal Peptide Synthase, LAP is Linear Azole/azoline-containing Peptide.

**Table 4:**
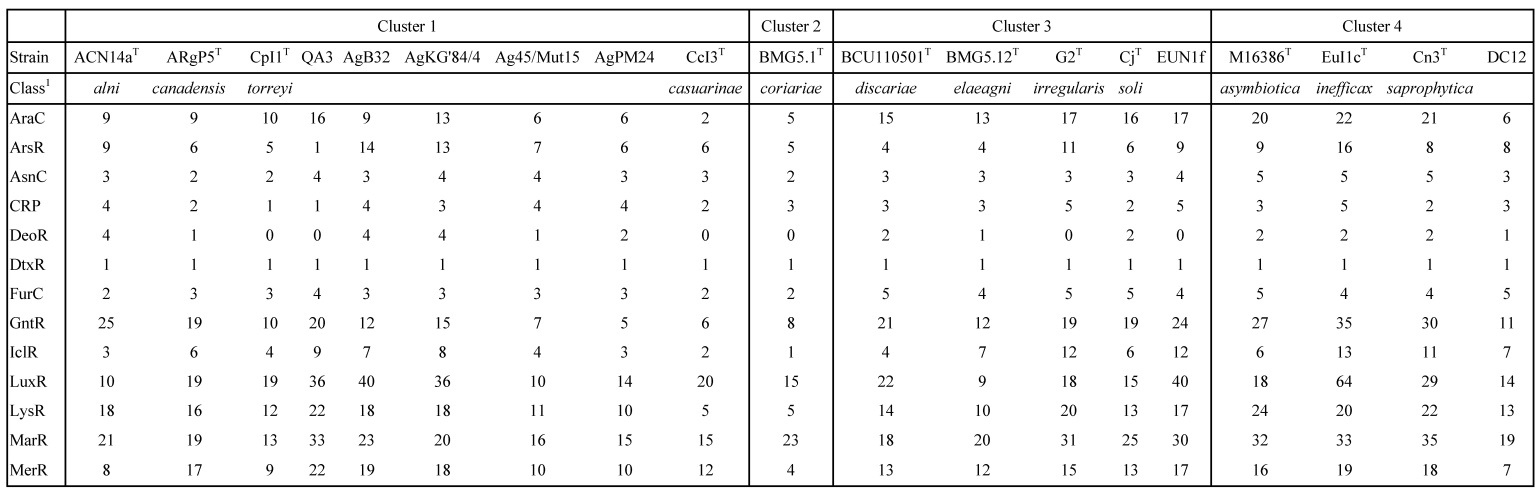
Number of transcriptional regulators of *Frankia* strains AgB32 and AgKG'84/4 compared to those of type strains of *Frankia* species in clusters 1 to 4

^1^class: **AraC**: arabinose regulator; **ArsR**: arsenic resistance; **AsnC**: asparagine synthase regulator; **CRP**: cyclic AMP receptor protein (catabolite repression); **DeoR**: deoxyribonucleoside synthesis operon regulator; **DtxR**: diphtheria toxin repressor; **FurC**: ferric uptake regulator; **GntR**: gluconate regulator; **IclR**: isocitrate lyase regulator; **LuxR**: quorum-sensing luminescence regulator; **LysR**: lysine regulator; **MarR**: Multiple antibiotic resistance regulator; **MerR**: mercury resistance regulator; **TetR**: Tetracycline repressor; **WhiB**: regulation of morphological differentiation.
